# Stress-Induced Graves Disease: Spontaneous Recovery After Stress Relief

**DOI:** 10.1210/jendso/bvad157

**Published:** 2023-12-13

**Authors:** Jeresa I A Willems, Daan J L van Twist, Robin P Peeters, Guy J M Mostard, Roderick F A Tummers-de Lind van Wijngaarden

**Affiliations:** Department of Internal Medicine, Zuyd Thyroid Center, Zuyderland Medical Center, 6162 BG Sittard-Geleen, The Netherlands; Department of Internal Medicine, Zuyd Thyroid Center, Zuyderland Medical Center, 6162 BG Sittard-Geleen, The Netherlands; Department of Internal Medicine, Academic Center for Thyroid Diseases, Erasmus Medical Center, 3015 GD Rotterdam, The Netherlands; Department of Internal Medicine, Zuyd Thyroid Center, Zuyderland Medical Center, 6162 BG Sittard-Geleen, The Netherlands; Department of Internal Medicine, Zuyd Thyroid Center, Zuyderland Medical Center, 6162 BG Sittard-Geleen, The Netherlands

**Keywords:** Graves’ disease, stress, stress relief, remission

## Abstract

**Purpose:**

Emotional stress is a precipitating factor for Graves disease (GD). However, the influence of stress relief on the course of GD is unknown. Here, we present a series of patients diagnosed with stress-induced GD in whom stress relief alone led to remission of GD.

**Cases:**

We report on 11 patients in whom hyperthyroid symptoms started just after severe emotional stress. All patients had suppressed thyroid-stimulating hormone (TSH) levels and elevated free thyroxine (FT4; 22.2–49.5 pmol/L) and TSH-receptor antibody (TRAb; 0.57–40 U/L) levels and were subsequently diagnosed with stress-induced GD. However, all patients declined antithyroid drug treatment. Surprisingly, clinical and biochemical remission was observed in 9 out of 11 patients after 1 to 3 and 2 to 7 months of self-reported stress relief, respectively. Five patients showed long-lasting remission (median follow-up 2.3 years). In 4 patients, remission was initially achieved, but GD relapsed 1 to 4 years afterwards. In 2 patients, treatment with antithyroid drugs was initiated because of rapidly increasing FT4 levels. Baseline FT4 and TRAb levels tended to be higher in patients who did not achieve remission. Furthermore, patients without long-lasting remission were more frequently known to have prior thyroid disease.

**Conclusion:**

We report on a series of patients with stress-induced GD in whom stress relief alone led to remission of GD (thus without antithyroid drugs). This may indicate that clinicians could consider stopping antithyroid drug treatment or at least shortening the treatment period after stress relief in patients with stress-induced GD.

Emotional stress is a precipitating factor for Graves disease (GD) [1]. However, the influence of stress relief on the course of GD is unknown. Here we report on a series of patients who (initially) declined antithyroid drug treatment for stress-induced GD. Interestingly, the majority of patients achieved remission with stress relief alone (thus without antithyroid drugs).

We report on 11 patients diagnosed with GD between 2016 and 2021 in whom hyperthyroid symptoms started after a stressful life event, such as death or illness of relatives (Table 1). All patients had suppressed thyroid-stimulating hormone (TSH) levels, elevated free thyroxine (FT4) levels and TSH-receptor antibodies (TRAb). In 3 patients with only slightly elevated TRAb levels, diagnosis was confirmed by thyroid scintigraphy. All patients declined antithyroid drug treatment because they attributed their complaints to emotional stress. Five patients were temporarily (1-6 months) treated with beta-blockers (4 patients with metoprolol and 1 patient with bisoprolol). Surprisingly, improvement of hyperthyroid symptoms (clinical remission) together with gradual normalization of serum TSH and FT4 levels (biochemical remission) was observed in 9 out of 11 patients. Clinical and biochemical remission was observed 1 to 3 and 2 to 7 months after stress relief, respectively. We considered the moment that stress relief was reported during an outpatient clinic visit, as the time that stress relief started. Five patients showed durable remission (median follow-up 2.3 years) (Fig. 1). In 4 patients, remission was initially achieved, but GD relapsed 1 to 4 years afterward (Fig. 2). In 2 patients, treatment with thiourea derivatives was initiated after 5 and 13 weeks because of rapidly increasing FT4 levels (Fig. 3). No differences between the patients who achieved remission and the patients who did not achieve remission were observed with respect to patient characteristics (including gender, age, smoking habits, amount and nature of comorbidities, family history of thyroid disorders, nature and weight of the experienced stressful life event), baseline TSH levels, and symptoms at first presentation. Patients who achieved transient remission or who did not achieve remission were more frequently known to have prior thyroid disease (subclinical hypothyroidism or prior episode of GD). Baseline FT4 and TRAb levels tended to be higher in patients who did not achieve remission. Furthermore, beta-blockers were more frequently prescribed and for longer duration in the patients who reached long-lasting remission. None of our patients had thyroid eye disease or large goiter, hence this could not be studied in our case series. Moreover, no cardiovascular events were observed in any of the patients during follow-up.

**Figure 1.**
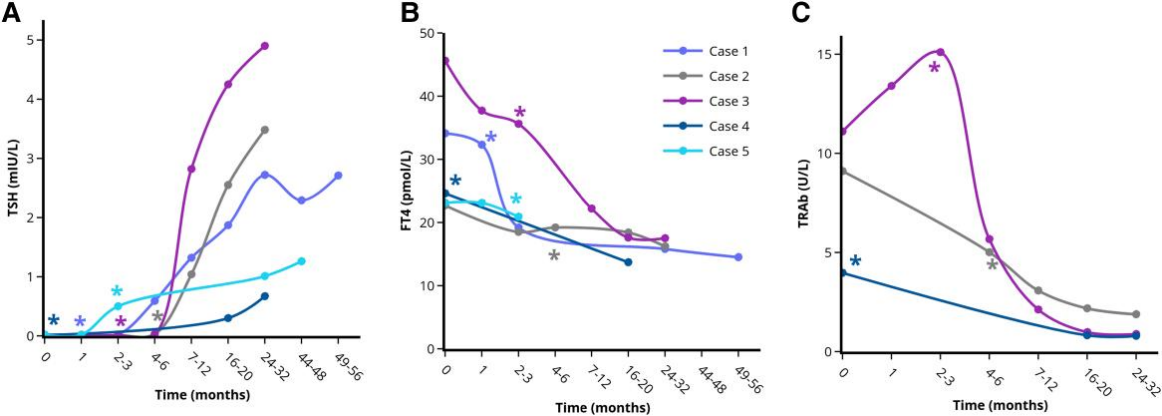
Course of serum TSH (A), FT4 (B), and TRAb (C) levels in the patients with long-lasting remission of GD after stress-relief. *Indicates the moment of self-reported stress-reduction on the outpatient clinic. Each case is represented by a unique colored line. Only cases with multiple TRAb measurements are included in Fig. 2C. Single (1-2) TRAb values are included in Table 1. Abbreviations: FT4, free thyroxine; TRAb, TSH-receptor antibodies; TSH, thyroid stimulating hormone. Figures were designed using the Figlinq platform (https://figlinq.com/).

**Figure 2.**
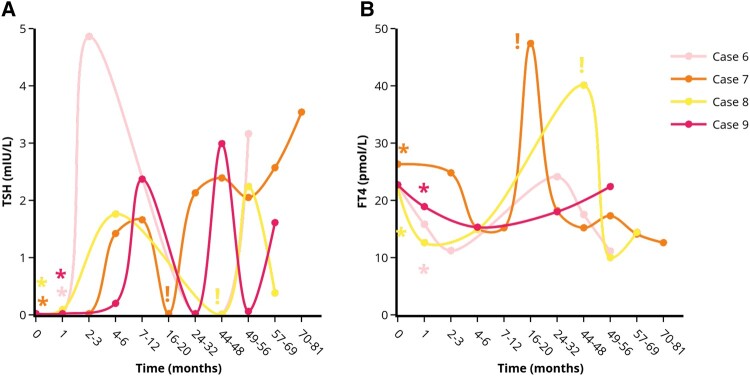
Course of serum TSH (A) and FT4 (B) levels in the patients with transient remission of GD after stress-relief. *Indicates the moment of self-reported stress-reduction on the outpatient clinic. !Indicates the moment antithyroid drug treatment was started. Each case is represented by a unique colored line. Abbreviations: FT4, free thyroxine; TSH, thyroid stimulating hormone. Figures were designed using the Figlinq platform (https://figlinq.com/).

**Figure 3.**
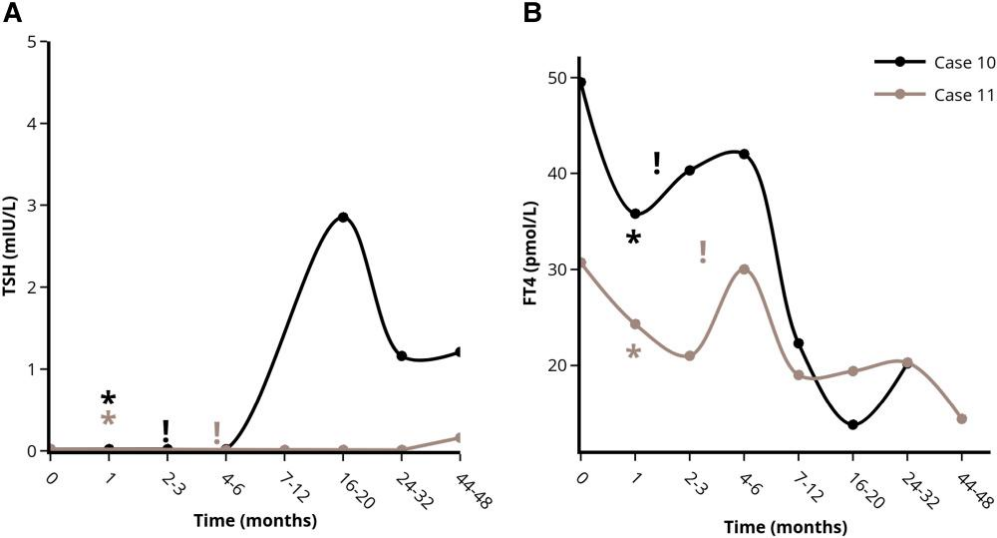
Course of serum TSH (A) and FT4 (B) levels in the patients without remission of stress-induced GD after stress-relief. *Indicates the moment of self-reported stress-reduction on the outpatient clinic. !Indicates the moment antithyroid drug treatment was started. Each case is represented by a unique colored line. Abbreviations: FT4, free thyroxine; TSH, thyroid stimulating hormone. Figures were designed using the Figlinq platform (https://figlinq.com/).

**Table 1.** Patients who initially declined antithyroid drug treatment for stress-induced Graves’ disease in Zuyderland Medical Center

Case	Sex	Age (years)	Stressful life event	TSH (mIU/L)	FT4 (pmol/L)	TRAb at diagnosis (U/L)	Time from stress relief until remission (weeks)		Remission (months from remission until recurrence)	TRAb during FU*^a^* (U/L)	Follow-up time (months)*^b^*	Beta-blocker use (weeks)	TSH during FU*^a^* (mIU/L)	Pre-event TSH (mIU/L) (years prior to GD)
							Clinical*^c^*	Biochemical*^d^*						
1	F	56	Death sister	<0.02	34.1	0.57*^e^*	4	12	Yes	?	48	Metoprolol (15)	2.71	1.30 (4)
2*^f^*	F	64	Hospitalization husband	<0.01	22.7	9.1	0	13	Yes	1.88	15	—	3.48	4.02 (2)
3*^g,h^*	F	68	Life-threatening illness husband	<0.01	45.6	11.10	13	29	Yes	0.88	12	Metoprolol (21)	4.90	3.92 (3)
4	F	71	Dementia husband	<0.01	24.6	3.97*^e^*	0	16	Yes	0.82	28	Metoprolol (25)	0.67	0.97 (0.5)
5	F	49	Relationship problems	<0.02	23.1	0.66*^e^*	0	12	Yes	0.44	43	—	1.26	1.02 (2)
6*^g^*	F	48	Anorexia nervosa daughter	<0.02	22.7	2.44	9	9	Recurrence (27)	?	Lost to FU	Propranolol (2) Metoprolol (5)	3.16	1.73 (1)
7*^f^*	F	42	Work-related problems	<0.02	26.3	4.45*^e^*	7	15	Recurrence (12)	?	Lost to FU	—	3.54	3.83 (1)
8	F	48	End of relationship	<0.02	22.2	0.92*^e^*	5	18	Recurrence (48)	?	54	—	0.38	?
9*^g^*	F	63	Death mother	<0.02	22.7	?	8	21	Recurrence (24)	?	55	—	1.61	2.39 (1)
10*^g^*	F	67	Death husband	<0.02	49.5	9.82	—	—	No	6.25	28	Propranolol (1) Bisoprolol (4)	1.21	1.65 (2)
11*^f^*	F	72	Death daughter	<0.02	30.7	>40.0*^e^*	—	—	No	8.56	Active treatment	—	0.16	10.57 (1)

Reference intervals (laboratory Zuyderland Medical Center): TSH: 0.27–4.20 mIU/L; FT4: 11.0–22.0 pmol/L; TRAb: < 0.55 U/L.

“—” indicates not performed or not used. “?” indicates unknown.

Abbreviations: F, female; FT4, free thyroxine; FU, follow-up; GD, Graves disease; M, male; TRAb, TSH-receptor antibodies; TSH, thyroid-stimulating-hormone.

^*a*^During the last assessment (if available).

^*b*^Follow-up time for patients with long-lasting and transient remission: biochemical remission after stress relief till last available TSH. Follow-up time for patients with no remission: biochemical remission after treatment till last available TSH.

^*c*^Clinical remission: the moment hyperthyroid symptoms were absent.

^*d*^Biochemical remission: the moment TSH and FT4 levels reached normal levels.

^*e*^GD diagnosis confirmed by thyroid scintigraphy.

^*f*^Prior subclinical hypothyroidism (not treated).

^*g*^Recurrence of GD.

^*h*^Subclinical hypothyroidism during follow-up (not treated).

To the best of our knowledge, this is the first report on remission of stress-induced GD after stress relief alone. Previous studies demonstrated that psychotherapy or benzodiazepines given concurrently with antithyroid drug treatment led to earlier remission and lower recurrence rates [1-3], but we found no studies on the effect of stress relief alone.

Stress may induce GD due to increased levels of glucocorticoids, catecholamines, and pro-inflammatory cytokines, leading to hyperactivity of the immune system. While glucocorticoids in pharmacological doses are known for their immunosuppressive effects, endogenous, physiological doses of glucocorticoids, released during stress, are thought to have immunomodulatory effects [4]. We hypothesize that stress relief restores the activity of the immune system back to normal. Yet, as it is difficult to quantify and determine the time of onset and relief of stress [1], it remains difficult to prove a causal relationship.

We considered several alternative explanations for our observation, such as thyroiditis or medication effect. First, for the patients that did not have radiographic confirmation of GD or pathognomonic features of GD on examination (such as thyroid bruit or ophthalmopathy), it is possible that they had thyroiditis instead, which would be extremely rare in the setting of TRAb positivity. Second, we considered influence of other medication unlikely. Three patients with long-lasting remission were temporarily (3-6 months) treated with metoprolol which lacks any antithyroid or immunosuppressive effects. Codaccioni et al showed that long-lasting remission of GD was achieved in 8 out of 26 patients after propranolol treatment only [5]. Propranolol inhibits the peripheral metabolism of thyroxine (T4) to triiodothyronine (T3), leading to a fall in serum T3 concentrations. However, for metoprolol such an effect is not described. Moreover, the authors noticed lower baseline free triiodothyronine (FT3) concentrations in the 8 patients who achieved remission but did not observe a fall in serum FT3 concentrations in the first weeks of propranolol treatment. They therefore considered a direct effect of propranolol on the remission of GD improbable and attributed the remission to the natural course of GD [5]. In our patients, we observed a clear temporal relationship between onset and relief of stress and the course of GD. Furthermore, none of our patients used dietary supplements that could explain GD remission [6].

Future studies are needed to confirm our observation and to identify subgroups of patients with stress-induced GD in whom treatment could be stopped or shortened after stress relief. We do not recommend this approach in patients with moderate to severe, active thyroid eye disease since the eye disease could worsen if hyperthyroidism is not promptly treated. Moreover, in a previous study, large goiters and/or a previous episode of hyperthyroidism were risk factors associated with a lower chance of remission [5]. Finally, as remission did not occur in our patients with very high TRAb and FT4 levels, this might be another risk factor for failure of this approach.

In conclusion, we report a series of patients in whom remission of stress-induced GD occurred after stress relief alone, without antithyroid drugs. This may indicate that clinicians could consider stopping antithyroid drug treatment or at least shortening the treatment period after stress relief in patients with stress-induced GD.

## Data Availability

Original data generated and analyzed during this study are included in this published article or in the data repositories listed in References.
